# Graduates from a traditional medical curriculum evaluate the effectiveness of their medical curriculum through interviews

**DOI:** 10.1186/1472-6920-9-64

**Published:** 2009-10-26

**Authors:** Simon Watmough, Helen O'Sullivan, David Taylor

**Affiliations:** 1 Centre for Excellence in Developing Professionalism, School of Medical Education University of Liverpool, Cedar House, Ashton Street, Liverpool, L69 3GE. UK

## Abstract

**Background:**

In 1996 The University of Liverpool reformed its medical course from a traditional lecture-based course to an integrated PBL curriculum. A project has been underway since 2000 to evaluate this change. Part of this project has involved gathering retrospective views on the relevance of both types of undergraduate education according to graduates. This paper focuses on the views of traditional Liverpool graduates approximately 6 years after graduation.

**Methods:**

From February 2006 to June 2006 interviews took place with 46 graduates from the last 2 cohorts to graduate from the traditional Liverpool curriculum.

**Results:**

The graduates were generally happy with their undergraduate education although they did feel there were some flaws in their curriculum. They felt they had picked up good history and examination skills and were content with their exposure to different specialties on clinical attachments. They were also pleased with their basic science teaching as preparation for postgraduate exams, however many complained about the overload and irrelevance of many lectures in the early years of their course, particular in biochemistry. There were many different views about how they integrated this science teaching into understanding disease processes and many didn't feel it was made relevant to them at the time they learned it. Retrospectively, they felt that they hadn't been clinically well prepared for the role of working as junior doctor, particularly the practical aspects of the job nor had enough exposure to research skills. Although there was little communication skills training in their course they didn't feel they would have benefited from this training as they managed to pick up had the required skills on clinical attachments.

**Conclusion:**

These interviews offer a historical snapshot of the views of graduates from a traditional course before many courses were reformed. There was some conflict in the interviews about the doctors enjoying their undergraduate education but then saying that they didn't feel they received good preparation for working as a junior doctor. Although the graduates were happy with their undergraduate education these interviews do highlight some of the reasons why the traditional curriculum was reformed at Liverpool.

## Background

Many medical schools around the world have reformed their medical curricula in recent years [1]. The UK, in particular has seen reforms in response to *Tomorrow's Doctors *[2,3]. There have been studies examining the impact of curriculum reform on the first year post graduation [4-11] and various quantitative studies have asked graduates retrospectively to evaluate their undergraduate education, [12-16] but there have been few qualitative studies asking graduates in retrospect to evaluate their undergraduate medical education. Whilst there were good reasons for reforming traditional curricula, few studies in the UK have examined traditional graduates' attitude to their undergraduate medical education prior to curricula being reformed. Therefore, it can be argued that there was still an imbalance in the literature pertaining to the reasons for curriculum reform. This paper aims to redress the balance by discussing interviews held with medical graduates from a traditional course approximately 6 years after graduation.

The interviews stem from a project that has been running since 2000 called "The Liverpool Medical Curriculum Evaluation Project". In 1996 Liverpool reformed its medical programme from a traditional lecture-based course to a community-based, integrated, problem based learning curriculum. The project involved evaluating the impact of curriculum reform by gathering views on the content of the reformed curriculum and examining the perceived competencies of the final 2 cohorts of the traditional curriculum and the first two cohorts of the reformed medical curriculum working as first year graduates. The initial results showed that the graduates from the reformed Liverpool curriculum were better prepared for the first post graduate year [4-6].

The more important measure however, is derived from examining the impact of curriculum reform beyond the first postgraduate year, after the graduates had made their career decisions. Six years as a postgraduate enables close enough recall of their graduation for the participants still to be able to reflect on their undergraduate programme but gave them enough postgraduate experience to make a significant evaluation of the effect of their education. This study aims to build on the previous work undertaken with these cohorts in their first postgraduate year and examines how effective traditional graduates perceived their undergraduate medical education was 6 years after graduation.

### The Liverpool Traditional Curriculum

The Traditional Curriculum was based on passive learning styles which comprised a 5 term pre-clinical course followed by a 9-term clinical course with little formal integration between the 2 parts of the curriculum. In the first five terms students undertook an intensive series of lectures and practicals in biochemistry, biology, biostatistics, genetics, anatomy, physiology, psychology and pathology. Students then learned undertook lectures and clinical placements in separate blocks in general medicine (including care for the elderly), general surgery (including orthopaedics) obstetrics and gynaecology (O & G), psychiatry, Ear Nose and Throat (ENT), child health, dermatology, cardiology, ophthalmology, neurology, haematology, pharmacology and a three week placement in General Practice (GP). Examinations took place during both sections of the course culminating in final exams at the end of 5^th ^year in medicine, surgery and O & G. There were no formal communication or clinical skills classes.

## Methods

Ethical approval was sought and gained from the National Health Service (NHS) COREC Liverpool Research Ethics Committee to contact the graduates for their consent to take part in this project. All UK doctors have a statutory duty be registered with the General Medical Council (GMC) in order to practice medicine. In September 2005 the GMC was contacted to supply registration numbers and contact addresses for 310 students who graduated in 1999 and 2000 (the final two cohorts to graduate from the traditional course). Details of 25 were unavailable, possibly because they had changed name (marriage) or were no longer registered with the GMC.

During autumn 2005, graduates were sent a letter and consent form inviting them to take part in both the questionnaire and interview parts of the project. Seventy eight graduates volunteered to take part in an interview. They were contacted three times via email from SW. If it proved impossible to arrange a mutually convenient time they were not contacted again. A total of 46 interviews, lasting 30-40 minutes took place between January and June 2006 so all participants had been working as postgraduate doctors for between 6 and 7 years.

There were 25 interviews with doctors who had graduated in 1999 and 21 interviews with graduates who had graduated in 2000 (n = 46), 26 female, 20 male. 16 interviewees were physicians, 13 were General Practitioners (GPs), 9 were surgeons, 6 psychiatrists and 2 anaesthetists with 3 interviewees taking time out of their training to undertake full time research. 36 of the interviews took place face to face either in the hospital, GP surgery, home of the interviewee or office of SW and ten interviews for those doctors who lived outside the Liverpool area took place via telephone.

### Analysis

The questions were based on the questions used with the focus groups which were held with these doctors as first year postgraduates and consultants who supervised them, during the early stages of the evaluation project [17-19] and the expectations of competencies of doctors according to the GMC [2]. These prior interviews and focus groups ascertained that doctors were happy to answer these kinds of questions about their undergraduate education. The interviewees had already returned questionnaires prior to these interviews taking place and from the responses to the questionnaire it was clear that these doctors were able to assess their undergraduate course. The interviewer was a non-clinician researcher (SW), who, prior to the interviews, was generally unknown to the interviewees. This, together with him not being in a management position within the University or the NHS reduced the possibility of bias [20] during the interviews. The interviews were tape-recorded and then transcribed *verbatim *by SW. The analysis was based on the framework approach [21] which allows the objectives of the research to be determined prior to data collection. These prior objectives were covered in the basic questions to all interviewees which included: how relevant was your basic science knowledge; how did you learn your communication skills; how well prepared were you to work as a junior doctor; were you well trained in history and examination skills; did you graduate with the necessary research skills to train as a postgraduate; did you receive adequate training undertaking practical procedures on patients; what were the strengths and weaknesses of your course; is there anything else you would like to add about your time as an undergraduate?

The framework approach involves clear stages of data analysis which were applied to the analysis of these interviews: familiarisation; identifying a thematic framework; indexing; charting; mapping and interpretation. The tapes and transcripts were re-listened to and re read for familiarisation. The thematic framework was then identified by examining the priori issues (in this case the questions) and issues raised by the interviewees. The data was clearly coded and the text was indexed by using descriptors alongside various passages in the transcriptions. The data was then charted alongside the appropriate part of the thematic framework and finally, the charts were mapped to explore associations between the themes and examine the original research objectives and emerging themes. The transcripts were originally analysed by SW using steps identified above [21,22]. The other authors of this paper independently read through the transcriptions and offered their own views on the themes emerging from the priori questions and emerging themes which were then cross-referenced with the codes and analysis of SW in order to reduce biases and validate the findings. There were no major differences in the views of the co-authors with those of SW of the themes and emerging issues. Using the framework approach meant that all the themes we considered important were covered in the interviews which helped gain saturation of themes. Kvale [23] has written that approximately 15 - 20 interviews with a group of people with similar backgrounds is often enough to gain saturation of themes so interviewing 46 doctors ensured no important views were missed out and that any potential differences between the two cohorts could be noted (although no differences between then two cohorts did emerge). The final questions, "what were the strengths and weaknesses of your course?" and "is there anything else you would like to add?" ensured respondents could identify issues which were important to them which may not have been covered previously in the interviews.

## Results

### Knowledge base

The doctors discussed whether they learned the required basic science knowledge base when they graduated and how much was relevant to how they practised 6 years after graduation. Perhaps, unsurprisingly there were mixed views on this and some of the views were very much specialty dependent. For example, some of those who became surgeons wanted more anatomy teaching, but the graduates who became GPs or psychiatrists wanted less anatomy teaching.

As a whole they did feel they graduated with a good science knowledge base and despite stating that they covered a lot of subjects in the pre-clinical part of the course which gave a good background knowledge there were complaints that they had learned too much irrelevant knowledge in those areas. For many, the first two years of the course were "dry" and "uninspiring" with biochemistry in particular being cited.

"I did feel with the biochemistry that we did too much and that was laboured more than it should have been"

"some of the lectures were very good, excellent, but some were rubbish...I remember sitting in some being thoroughly confused..."

"The lectures served me well, I didn't need to know it all but it gave me a good grounding"

The majority also felt that although they had forgotten a lot of what they had learned it was useful to have that some of that background when revising for postgraduate Royal College exams. For many of the interviewees, though knowing the science for postgraduate exams was more useful than knowing it for diagnosing patients.

"..it was logical and although a lot of it didn't apply to being a doctor you got the bits that did apply and could build on that, certainly when revising for postgraduate exams."

The interviewees were split about when they tied their science understanding together to understand disease processes. None of the interviewees managed to grasp this in the pre-clinical course, but then they were split almost 3 ways whether it came together for them during the early part of the clinical course, final exams or when working in the first postgraduate year.

### History and examination skills

The interviewees felt that they had received a good grounding in taking history and examination skills and many of them still referred back to the tips they picked up as undergraduates when they examined patients working as doctors.

"It is drilled into you like nothing else"

"I got it from an early time, the first medical attachment I did. It was repeated and repeated. I presented to the registrar and consultant until it stuck. It was good."

They felt that the first general medical and surgical placements and the long final year placements in medicine and surgery were useful for gaining these skills. These attachments were also seen as most beneficial for learning about differential diagnoses - which all the graduates felt they had a good grounding in. Some, though, did feel intimidated by learning the skills with consultants on the wards straight after the pre clinical course.

"I found it a bit overwhelming doing learning it on the wards in my first attachments."

Despite this, all interviewees did feel that they were very competent in these areas when they graduated.

### Communication skills

The vast majority of graduates felt they were competent communicators despite not having specific communication skills classes.

"I think I do have good communication skills, but I don't think I got them from medical school, I think I got them from my parents."

They felt they had managed to acquire the necessary communication skills simply by observing other doctors on clinical placements as undergraduates and didn't feel they needed tuition.

"you can just pick up your skills by observing senior doctors, what was good...what was bad and pick the style you would like in the future."

Only a small number of interviewees felt tuition would have been beneficial and mostly these were GPs. Some of the interviewees linked communication skills solely with being able to take a competent history rather than, breaking bad news or conversations with patients' families for example, which perhaps illustrated a lack of perception about communication skills.

### Research skills

Apart from the 5 interviewees who had taken intercalated degrees none of the graduates indicated that they had received any experience in learning about research skills.

"no, it just wasn't like that, I don't think we thought about things like that really."

" I didn't get an understanding of audit or of research.. there was very little of that which is a disadvantage now because in postgraduate medicine it is something that is seen as very important now."

They did feel that students who undertook a PBL education would have these skills.

"that is one of the things I have been impressed with the undergraduate courses now - they can research and they have their heads around understanding publications much sooner."

### Practical skills/preparedness for role of junior doctor

Although it was 6 years since these doctors graduated they were asked about how well prepared they had been to work as first year medical graduates and what their tuition in gaining the practical skills associated with the role were like. The consensus was that they hadn't been well prepared in this area.

"I didn't feel well prepared at all, I felt like a scared rabbit for the first 6 months."

"I think the biggest downfall of the traditional course is that it doesn't prepare you for the first day at work"

"when I got lumped on the ward I didn't have a clue what to do....where to start.. whether to examine them, whether to take bloods or observations.. I didn't know how to fill in an x ray form.. prescribe paracetemol...it was a horrible few weeks.."

There were low expectations of how well an undergraduate curriculum could prepare graduates for work, anyway.

"I don't think anything can prepare you... but you learn on the job, pick it up and everyone is in the same boat.."

Many interviewees said that they spent their final year learning what they needed to know for their exams rather than what they needed to know to begin postgraduate training.

### Strengths and weaknesses of the course

The interviewees did feel that the structure of the course was good and useful with the pre clinical/clinical divide and there was a good amount of clinical exposure. They believed that the course was "logical" and that they found it reassuring to be told what they needed to know through lectures, which clinical attachments they had to go on and there was some good bedside teaching.

"In essence we were spoon fed and everything we needed to know and it was perfect for me."

However, the fact there were two distinct pre clinical and clinical sections of the course was also seen as weakness as well. Many respondents cited the grounding in the basic sciences as a strength of the course but they felt they had learned too much irrelevant knowledge and many of the lectures were seen as boring.

"It was thorough and we covered a lot of ground but this was also a weakness as it was too thorough!"

The overwhelming weakness according to interviewees was lack of preparation to work as a first year postgraduate and, to a lesser degree, lack of exposure to research skills. The graduates were almost split down the middle over whether the amount of General Practice was enough or whether it should have been increased despite approximately a third of the doctors saying they had little idea about the relationship between primary and secondary care. The GPs in the study, though felt there were far too few community placements. However, despite the lack of preparation for their first job after graduation and knowledge "overload" they had enjoyed their course and the vast majority were glad they studied under the traditional curriculum rather than a reformed curriculum.

## Discussion

There were some potential limitations to this study. It is possible that the interviewees may have represented people who had either more positive or more negative views of their undergraduate education. However, a large number were interviewed to account for variations in experience and expectation, and to cover for the fact that the volunteers may have been people who were either particularly happy or unhappy with the course. However, there were no interviewees in the study who had only positive or negative views about their undergraduate education. Also, it is important to stress, all the graduates were comfortable to say what they felt was good and bad about the course for most of the questions there was a clear consensus. A limitation of the study could be that only 46 out of a possible 310 were interviewed. However, the interviews were representative of the gender ratio of the cohorts and covered a wide range of specialties. It is not clear how representative of the cohort as a whole the interviews were, as we do not have career details for all graduates. However, for the questionnaire part of the study [24] which represented 116 doctors and 37% of possible respondents, the respondents consisted of 21 surgeons, 41 physicians, 38 GPs, 11 psychiatrists and 5 graduates who were involved in full time research at the time the questionnaires were distributed. Therefore, the ratio of doctors who took part in this study is similar to the larger number of doctors who took part in the questionnaire survey which suggests the sample here may be representative of the cohort as a whole. Although more interviews could have been arranged, interviewing 46 graduates is more than the accepted amount for gaining consensus on a group of people with similar backgrounds where 10/15 can be seen as enough to gain saturation of themes [23].

Although these medical graduates were from just one university the traditional curriculum at Liverpool was based on traditional curricula which were standard not just in the UK, but elsewhere for over 100 years [2,25]. Therefore, these interviews offer a historical snap shot of the views of graduates who studied under a traditional course just prior to curricula in the UK being reformed after *Tomorrow's Doctors*. Despite the body of literature suggesting that undergraduate medical curricula should be reformed [2], few studies such as this one had been carried out on graduates from a traditional medical curriculum.

The graduates were generally happy with their course. Although this may be partly down to an understandable "nostalgia" to their student lives, even those who felt there should have been more General Practice or better preparation for the first postgraduate year were genuinely glad they had studied under their traditional curriculum. On the surface, given that fact that many were unhappy with aspects of their course such as excessive basic science teaching or lack of preparation for the role of being a junior doctor it may seem surprising that only a small number would have preferred to have studied under a different curriculum. However, it is the only course they had experienced and may have felt threatened or undermined by the introduction of a new curriculum whilst they were still in medical school. Also, the interviewees clearly stated what they felt were the strengths of the curriculum; a good variety of clinical attachments which gave the opportunity to gain history and examination skills; comprehensive covering of knowledge base which gave the graduates confidence when studying for postgraduate exams and the fact it was well "structured" and they felt they were told what they needed to learn.

Despite this, they underlined what had been identified as the fundamental weakness of traditional curricula at the time of curriculum reform. The graduates, whilst recognising the benefits of having structured science teaching, felt there were far too many lectures and had to learn irrelevant facts, particularly in biochemistry. They also cited how poorly prepared they had been for the first postgraduate year and how they had a lack of research skills. It can also be seen as worrying that a large number of them were happy to have been "spoon fed" when the aim of undergraduate medical should be to create independent practitioners [2,3].

Their views about their basic science knowledge implies a mismatch between learning a subject and then having the opportunity to apply that learning, especially because there were so many different views about when they tied their science understanding together to understand disease processes. This also seems to imply it was left to chance or to the individual rather than the teaching within the curriculum despite the feeling from the interviews that "structure" of the course was good and they felt they were told what they needed to know. Certainly, the interviewees indicated that most of the skills they required, including clinical skills and communication skills were picked up almost by accidental learning during the large amount of time on the wards, which included 26 weeks of medicine and surgery in the final year, rather than explicit clinical teaching. Many did mention that they had been busy learning for final exams rather than learning about their future role as a doctor. So despite a large amount of clinical exposure, because they weren't being directed to learn the skills they would need as first year postgraduates they didn't manage to learn them. Also, not having experienced specific communication skills classes, the majority of the interviewees struggled with the concept that communication skills could be taught. This does not correspond with the research evidence concerning the benefit of communication skills training [26,27]. The fact that traditional graduates didn't feel prepared for practice raises concerns about their own confidence when it came to treating patients in their first year after graduation.

## Conclusion

Although a traditional course is no longer in place at Liverpool these interviews still hold relevance. Despite *Tomorrow's Doctors*, many curricula in the UK do still have a "traditional" element to them, as do many medical curricula around the world. Including traditional graduates in the medical evaluation project has allowed a fuller picture of the impact of curriculum reform to be gauged since direct comparisons can be made with the graduates from the reformed curriculum [4-6]. Despite their comfort with the programme these interviews demonstrate some of the reasons why the traditional course was reformed at Liverpool and a new course introduced [28].

## Competing interests

The authors declare that they have no competing interests.

## Authors' contributions

SW conceived and designed the study, analysed and interpreted the data, drafted the article and approved the final version to be published. HOS and DT reviewed and analysed the data, contributed to the first and subsequent drafts and approved the final version to be published.

## Pre-publication history

The pre-publication history for this paper can be accessed here:



## References

[B1] Bleakley A, Brice J, Bligh J (2008). Thinking the post-colonial in medical education. Medical Education.

[B2] General Medical Council (1993). Tomorrow's Doctors Recommendations on Undergraduate Medical Education.

[B3] General Medical Council (2003). Tomorrow's Doctors Recommendations on Undergraduate Medical Education.

[B4] Watmough S, Ryland I, Garden A, Taylor D (2006). Educational supervisors' views on the competencies of pre registration house officers. British Journal of Hospital Medicine.

[B5] Watmough S, Ryland I, Garden A, Taylor D (2006). Preregistration house officer skill and competency assessment through questionnaires. British Journal of Hospital Medicine.

[B6] Watmough S, Garden A, Taylor D (2006). Does a new integrated PBL curriculum with specific communication skills classes produce Pre-Registration House Officers (PRHOs) with improved communication skills?. Medical Teacher.

[B7] Jones A, O'Neill P, McArdle P (2002). Perceptions of how well graduates are prepared for the role of pre-registration house officer: a comparison of outcomes from a traditional and integrated PBL curriculum. Medical Education.

[B8] Richardson I (1983). Consumer views on the medical curriculum: a retrospective study of Aberdeen graduates. Medical Education.

[B9] Prince K, Van Eijs P, Boshuizen H, Vleuton C Van der, Scherpbier A (2005). General competencies of problem-based learning (PBL) and non-PBL graduates. Medical Education.

[B10] Kaufman D, Mann K (1998). Comparing achievement on the Medical Council of Canada Qualifying Examination Part I of students in conventional and problem-based learning curricula. Academic Medicine.

[B11] Cave J, Goldacre M, Lambert T, Woolf K, Jones A, Dacre J (2007). Newly qualified doctors' views about whether their medical school had trained them well: questionnaire surveys. BMC Med Educ.

[B12] Antepohl W, Domeij E, Forsberg P, Ludvigsson J (2003). A follow up of medical graduates of a problem-based learning curriculum. Medical Education.

[B13] Hyppola H, Kumpusalo E, Virjo I, Mattilo K, Neittaanmaki L, Halila Kujala S, Luhtala Isokoski M (2000). Evaluation of undergraduate medical education in Finnish community-oriented and traditional medical faculties: a 10-year follow-up. Medical Education.

[B14] Hyppola H, Kumpusalo E, Virjo I, Mattilo K, Neittaanmaki L, Halila, Kujala S, Luhtala Isokoski M (2002). Improvement in undergraduate medical education: a 10-year follow up in Finland. Medical Teacher.

[B15] Schmidt H, Vermeulen L, Molen H van der (2006). Long term effects of problem-based learning: a comparison of competencies acquired by graduates of a problem-based and a conventional medical school. Medical Education.

[B16] Cohen-Schotanus J, Muijtjens A, Schönrock-Adema J, Geertsma J, Vleuten C van der (2008). Effects of conventional and problem-based learning on clinical and general competencies and career development. Medical Education.

[B17] Watmough S, Garden A, Taylor D (2006). Educational Supervisors evaluate the preparedness of graduates from a reformed UK curriculum to work as Pre-registration House Officers (PRHOs): A qualitative study. Medical Education.

[B18] Watmough S, Taylor D, Garden A (2006). Pre-registration house officers (PRHOs) give their views about studying under a reformed medical curriculum in the UK. Medical Education.

[B19] Watmough S, Garden A, Graham D Pre-registration house officers (PRHOs) assess their undergraduate medical education through focus groups. Proceedings of the Annual Conference of the Association for Medical Education in Europe (AMEE); 2002 August 29 - September 1; Lisbon, Portugal.

[B20] Norris N (1997). Error, bias and validity in qualitative research. Educational Action Res.

[B21] Ritchie J, Spencer L, Bryman A, Burgess RG (1994). Qualitative data analysis for applied policy research. Analysing Qualitative data.

[B22] Miles M, Huberman M (1994). Qualitative data analysis: an expanded sourcebook.

[B23] Kvale S (1994). Interviews An Introduction to Qualitative Research Interviewing.

[B24] Watmough S, Ryland I, Taylor D (2007). Using questionnaires to determine whether medical graduates career choice is determined by undergraduate or postgraduate experiences. Medical Teacher.

[B25] Bullimore David (1998). Study Skills and Tomorrow's Doctors.

[B26] Yedidia M, Gillespie C, Kachur E, Schartz M, Ockene J, Chepaitos A, Snyder C, Lipkin M (2003). Effect of Communications Training on Medical Student Performance. Journal of the American Medical Association.

[B27] Van Dalen J, Kerkhofs E, Van Knippenberg-Van Den Berg B, Can Den Hout H, Scherpbier A, Vleuten C Van Der (2002). Longitudinal and Concentrated Communication Skills Programmes: Two Dutch Medical Schools Compared. Advances in Health Sciences Education.

[B28] Bligh J, Parsell G (1995). The changing context of medical education. Postgrad Medical Journal.

